# Concurrent Hashimoto Thyroiditis, Graves’ Disease, and Papillary Thyroid Carcinoma: A Case Report

**DOI:** 10.3390/reports9030248

**Published:** 2026-08-01

**Authors:** Venera Berisha-Muharremi, Alberta Humolli, Jehona Telaku, Fisnik Kurshumliu, Reshat Mati

**Affiliations:** 1 Faculty of Medicine, University of Prishtina, 10000 Prishtina, Kosovo; venera.berisha@uni-pr.edu (V.B.-M.); telakujehona2@gmail.com (J.T.); fisnik.kurshumliu@uni-pr.edu (F.K.); matimed@yahoo.com (R.M.); 2 Department of Endocrinology, Clinic of Endocrinology, University Clinical Centre of Kosovo, 10000 Prishtina, Kosovo; 3 Department of Endocrinology, Poliklinika Endomedica, 10000 Prishtina, Kosovo; 4 Faculty of Medicine, University of Ljubljana, 1000 Ljubljana, Slovenia; 5 Institute of Anatomic Pathology, University Clinical Centre of Kosovo, 10000 Prishtina, Kosovo

**Keywords:** autoimmune thyroid disease, Graves’ disease, Graves’ orbitopathy, Hashimoto thyroiditis, papillary thyroid carcinoma

## Abstract

**Background and Clinical Significance**: The co-occurrence of Hashimoto thyroiditis (HT), Graves’ disease (GD), and papillary thyroid carcinoma (PTC) is an extremely rare event and offers a unique opportunity to examine how chronic autoimmune thyroid disease may interact with the process of thyroid cancer development. **Case Presentation**: We present the case of a 42-year-old female with long-standing autoimmune thyroid disorders who developed progressive Graves’-related orbitopathy that did not improve with corticosteroid therapy. Owing to persistent hyperthyroidism and worsening orbital symptoms, she underwent total thyroidectomy. A histopathological examination of the removed thyroid gland surprisingly found PTC developing within a background of chronic autoimmune inflammation. The patient’s postoperative course included stable thyroid hormone replacement, a significant decrease in serum levels of thyroid autoantibodies, and clinical improvement in her orbital symptoms following decompressive surgery, although partial visual impairment persisted. **Conclusions**: This case highlights how chronic thyroid autoimmunity may create a carcinogenic environment and emphasizes the importance of comprehensive histopathological assessment and multidisciplinary care for patients with complex autoimmune thyroid disease.

## 1. Introduction and Clinical Significance

Hashimoto thyroiditis (HT) and Graves’ disease (GD) are the two most common forms of autoimmune thyroid diseases, which share some similarities in their pathogenic mechanisms; however, they exhibit different clinical and immune profiles [1]. HT is characterized by lymphocytic infiltration, the presence of thyroid autoantibodies, and a gradual progression towards hypothyroidism [2]. On the other hand, GD is caused by thyroid-stimulating hormone (TSH) receptor-stimulating antibodies, typically causing hyperthyroidism and, in many patients, varying degrees of Graves’ orbitopathy (GO) [3,4]. The relationship among these diseases reflects the complexity of interactions among genetic, environmental, and epigenetic factors involved in autoimmune thyroid disease [5].

Recent evidence suggests that HT and GD represent different clinical phenotypes within the spectrum of autoimmune thyroid disease rather than entirely distinct disorders. Shared genetic susceptibility, immune dysregulation, and environmental triggers may contribute to phenotypic switching between hypothyroidism and hyperthyroidism in susceptible individuals [6,7].

Papillary thyroid carcinoma (PTC) is the most frequent type of thyroid malignancy [8,9]. Several studies have reported an association between autoimmune thyroiditis and PTC; however, whether this reflects a true biological relationship or a coincidental coexistence remains uncertain [10,11]. Although the association between Graves’ disease (GD) and thyroid carcinoma also remains controversial [12], recent evidence suggests that chronic immune activation, sustained cytokine production, oxidative stress, and alterations in the thyroid microenvironment may contribute to thyroid carcinogenesis. Nevertheless, whether GD independently increases the risk of PTC has not been established, and conflicting results have been reported across studies [10,11,12,13,14].

Because of the rarity of this coexistence, additional well-documented case reports remain important for improving the understanding of disease progression, diagnostic challenges, and optimal clinical management.

Although several reports have demonstrated an association between HT, GD and PTC individually, the combination of all three entities in a single patient is extremely rare. To our knowledge, only one such report was previously published, concerning a male patient [15]. Therefore, documenting the coexistence of the three entities in a woman presents an additional illustration of the clinical diversity of the disease and provides an additional opportunity to study the relationships between thyroid autoimmunity and carcinogenesis.

## 2. Case Presentation

### 2.1. Patient Information

This patient is a 42-year-old woman with a background of long-standing autoimmune thyroid disease. She had been followed since 2014 for GD and HT. She had been followed since 2014 for HT with hypothyroidism and previously documented elevated anti-thyroid peroxidase antibodies (anti-TPO), requiring long-term levothyroxine replacement therapy. Until February 2024, she was clinically stable on levothyroxine replacement (75 μg twice weekly and 50 μg on other days). In February 2024, she developed palpitations, tachycardia, and progressively worsening eyeball discomfort. She had no relevant family history and denied any smoking or alcohol use.

### 2.2. Clinical Findings

**Figure 1:**
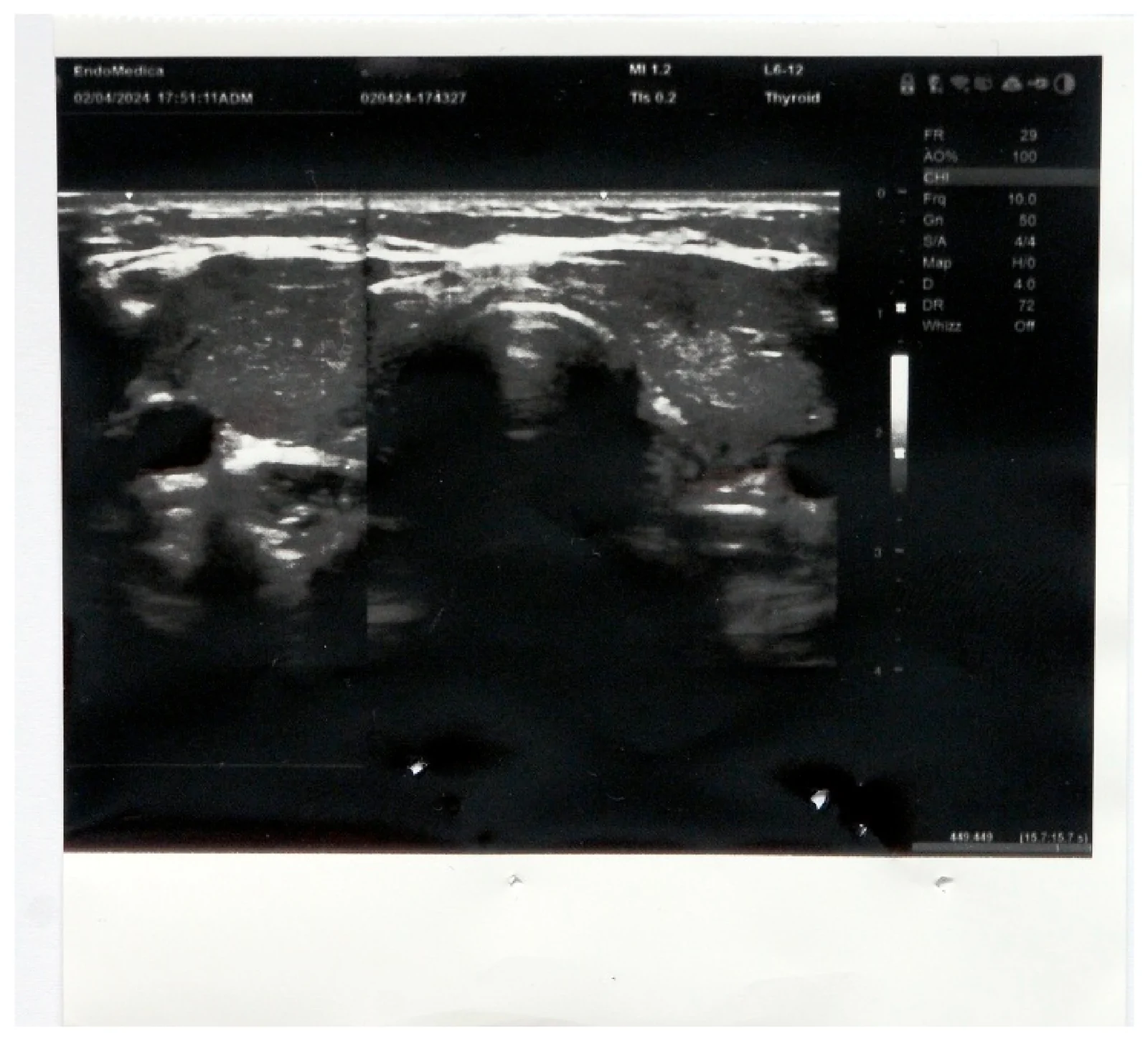
Neck ultrasonography (April 2024) showing enlarged thyroid lobes with heterogeneous hypoechogenic parenchyma, consistent with autoimmune thyroiditis. Isthmus: 1.2 × 0.37 × 2.6 cm.

On admission (March 2024), she appeared anxious and there was evidence of ocular pain with ocular motility disrupted. Heart rate (HR): 110 beats per minute (bpm); blood pressure (BP): 125/80 mmHg; normal temperature. Hertel exophthalmometry measurements: Bilaterally 26 mm consistent with proptosis. No cervical lymphadenopathy; cardiovascular and respiratory examination was unremarkable. Neck ultrasonography (April 2024) showed enlarged, heterogeneous, hypoechoic lobes with fibrotic bands and increased vascularity consistent with chronic autoimmune thyroiditis. No thyroid nodules or pathologically enlarged cervical lymph nodes were identified (Figure 1).

### 2.3. Diagnostic Assessment

Laboratory tests revealed suppressed TSH (<0.05 µIU/mL), raised free triiodothyronine (FT3), and free thyroxine (FT4), a notable increase in anti-TPO antibodies, positive thyroid-stimulating hormone receptor antibody (TRAb, 17.5 IU/L), mild elevation in C-reactive protein (CRP), borderline anemia and vitamin D deficiency (see Table 1). Given the presence of clinically evident thyroid-associated orbitopathy, neck ultrasonography was performed as part of the diagnostic evaluation.

Taking the clinical, laboratory, and imaging findings into consideration, the diagnosis of GD was based on the presence of overt hyperthyroidism, diffuse goiter, thyroid-associated orbitopathy, and positive TRAb in a patient with pre-existing HT. No malignancy was suspected prior to surgery.

### 2.4. Therapeutic Intervention

She discontinued levothyroxine and was treated with intravenous corticosteroids that tapered over six weeks from 40 mg to 5 mg. Additionally, she began taking methimazole (10 mg/day), propranolol (20 mg twice daily), atorvastatin (20 mg/day), selenium (200 µg/day), and pantoprazole (20 mg/day). Despite these treatments, the disease remained unstable with persistent thyrotoxicosis and worsening orbitopathy. Given the presence of progressive Graves’ orbitopathy requiring definitive treatment, total thyroidectomy was selected in accordance with current clinical recommendations [16]. As such, a total thyroidectomy was completed in May 2024 as definitive treatment for GD. Intraoperatively, both lobes were moderately enlarged without discrete nodules. Recurrent laryngeal nerves and parathyroid glands were preserved.

In October of 2024, she underwent bilateral orbital decompression with blepharoplasty involving lateral, medial, and inferior wall decompression with partial fat excision for continued progression of orbitopathy. Her postoperative treatment consisted of antibiotics, topical corticosteroids, lubricants, and NSAIDs.

### 2.5. Follow-Up and Outcomes

**Figure 2:**
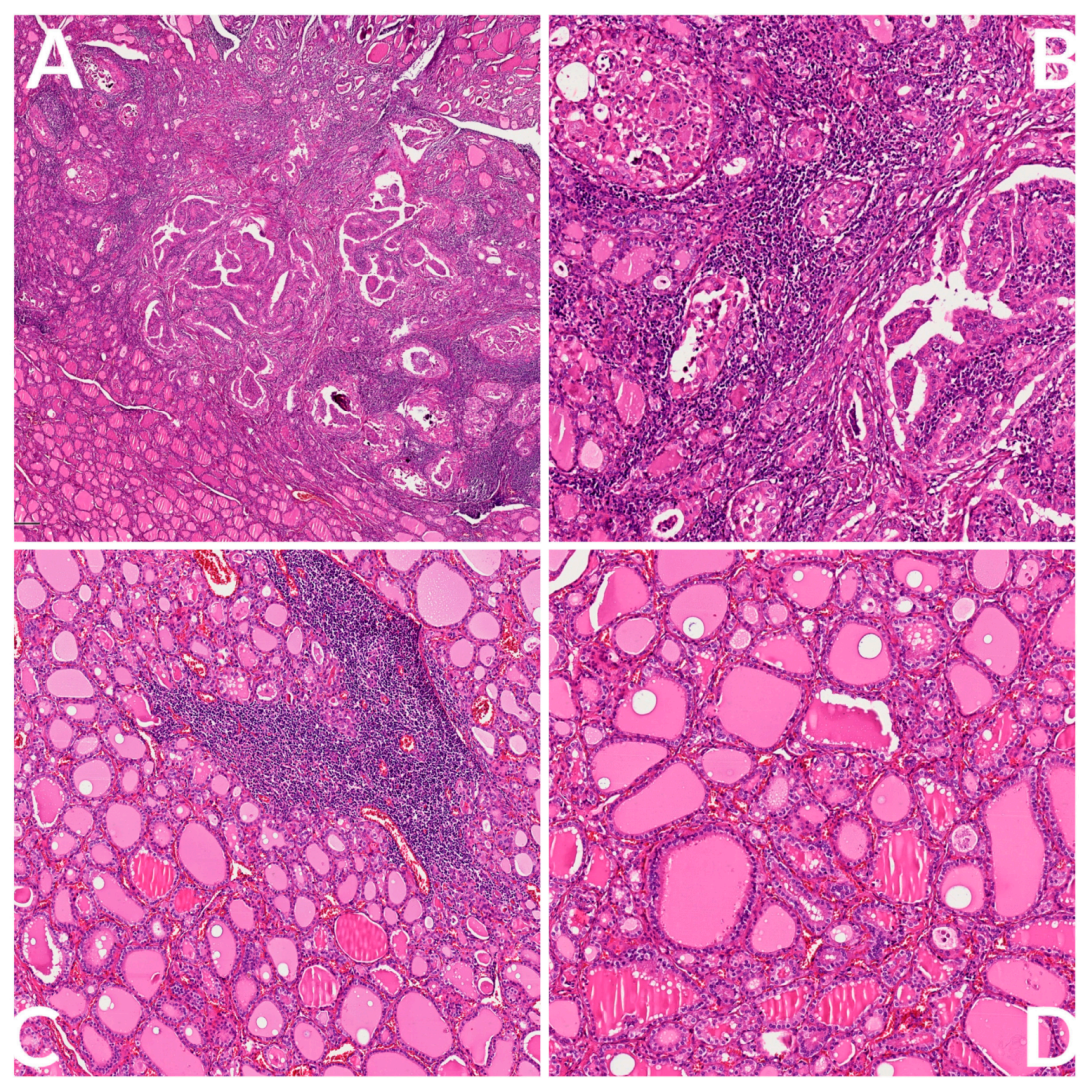
Histological sections showing unencapsulated papillary thyroid carcinoma surrounded by dense lymphoid infiltration (**A**,**B**) and adjacent hyperactive thyroid follicles with eosinophilic colloid and resorption vacuoles consistent with Graves’ disease (**C**,**D**). H&E staining; magnifications: 4× (**A**), 10× (**B**), 20× (**C**), and 40× (**D**).

Following total thyroidectomy, histopathological examination of her surgical specimen revealed an incidental 1.2 × 0.7 cm PTC located in the isthmus (pT1b pNx, R0), arising in a background of chronic autoimmune thyroiditis. Microscopic examination demonstrated characteristic PTC nuclear features without evidence of lymphovascular invasion. R0 indicated complete tumor resection with histologically negative surgical margins (Figure 2).

**Figure 3:**
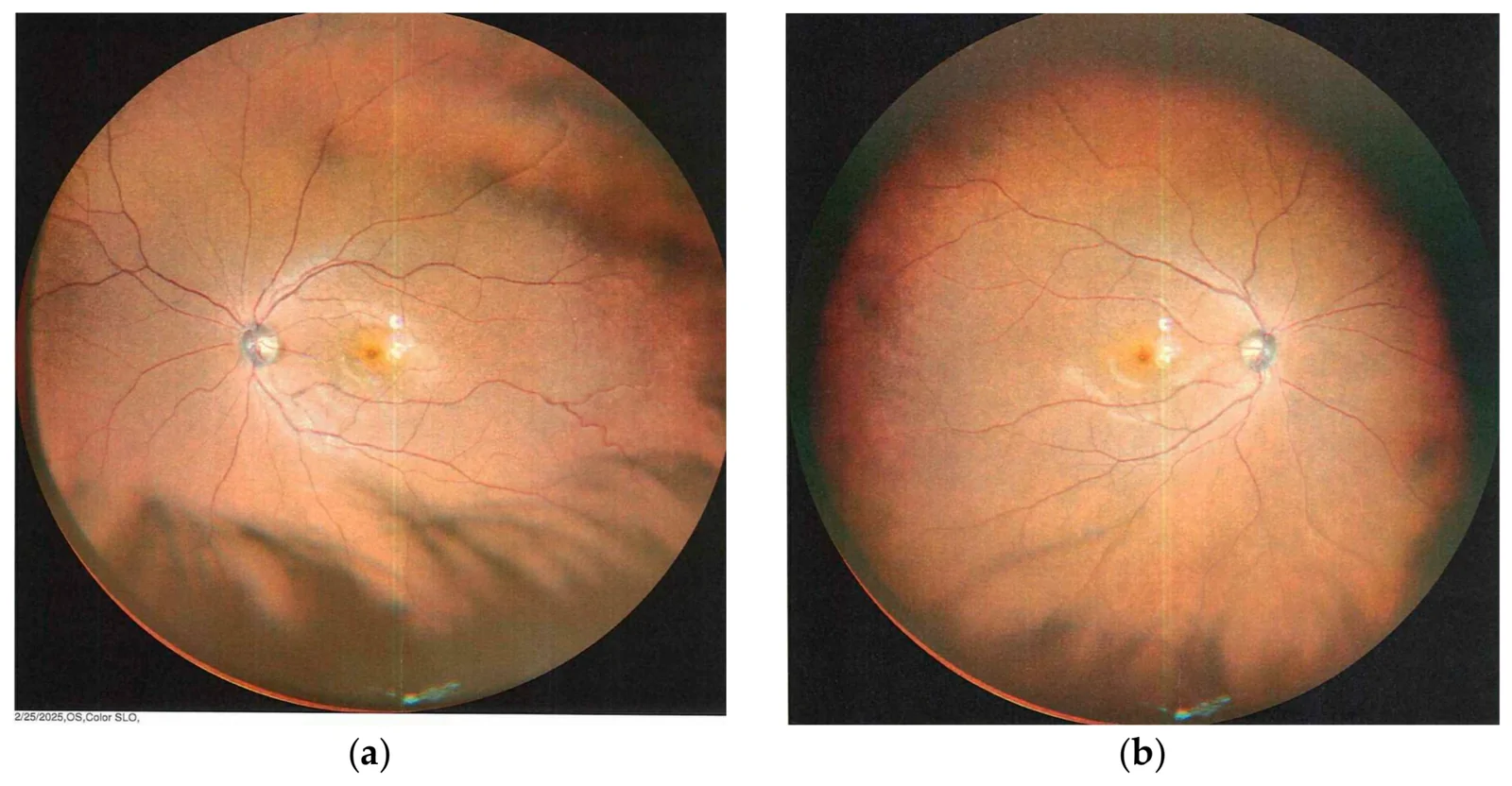
Fundus photographs after orbital decompression (February 2025): (**a**) Left eye; (**b**) right eye.

At follow-up in early 2025, the patient had stable thyroid function while receiving levothyroxine (125 μg on an alternating day basis), accompanied by a marked reduction in anti-TPO titers. Her ophthalmologic evaluation showed stable orbital structures but reduced visual acuities (right eye = 0.5, left eye = 0.21). Magnetic resonance imaging (MRI) confirmed adequate decompression without optic nerve involvement (Figure 3).

## 3. Discussion

Although the coexistence of GD, HT, and PTC may be underreported, only a limited number of well-documented case reports describing the simultaneous occurrence of all three conditions have been published [15]. Compared with the previously reported male patient [17], our case involved a female patient with severe and progressive GO refractory to corticosteroid therapy, ultimately requiring orbital decompression and blepharoplasty. Although papillary thyroid carcinoma may have represented an incidental finding in the background of HT, this case highlights the diagnostic and therapeutic challenges associated with the coexistence of these conditions. Moreover, PTC was not clinically suspected preoperatively and was diagnosed incidentally following thyroidectomy performed primarily for the management of uncontrolled hyperthyroidism and orbitopathy. Similar to the previously reported case, the coexistence of HT, GD, and PTC supports the hypothesis that chronic autoimmune inflammation may contribute to a pro-tumorigenic thyroid microenvironment. However, given the very limited number of reported cases, the precise pathogenic relationship among these entities remains to be elucidated. Therefore, we extend the literature by demonstrating this combination in a woman, emphasizing the need to consider all these entities together clinically and to perform a detailed pathological examination of all surgical specimens.

As demonstrated in several studies before, chronic autoimmune thyroiditis likely created a pro-tumor microenvironment that predisposed this patient to develop PTC [11,17]. The incidental discovery of carcinoma during the thyroidectomy, performed as definitive treatment for GD in the setting of progressive GO, highlights the ongoing controversy regarding the association between GD and thyroid cancer [12,16,18]. Furthermore, the mildly elevated CRP observed at presentation supports the presence of low-grade systemic inflammation accompanying active autoimmune thyroid disease. Although no published data are currently available regarding the prevalence of incidental PTC in Kosovo, autopsy studies from neighboring populations have reported occult papillary thyroid carcinoma in 5.6% of cases and thyroid carcinoma overall in 7.7% of cases [19]. These findings are consistent with a large meta-analysis of autopsy studies reporting a prevalence of incidental differentiated thyroid carcinoma ranging from approximately 4% to 11%, depending on the extent of histopathological examination [20]. Together, these data suggest that some incidental PTCs may represent coincidental findings rather than a causal association, supporting the possibility that the carcinoma identified in our patient was incidental rather than directly related to the coexistence of GD and HT.

PTC was discovered incidentally during thyroidectomy performed as definitive treatment for GD. Although incidental PTCs may represent clinically indolent lesions, this case highlights the importance of careful histopathological examination of all surgically removed thyroid specimens, even when surgery is not performed for suspected malignancy.

GO, found in 25–50% of GD patients, but severe in fewer than half, also influenced the patient’s course [21,22]. Corticosteroids were used in treatment and provided limited relief. Ultimately, thyroidectomy followed by orbital decompression were necessary [21], and both procedures significantly improved pain and appearance. However, complete restoration of vision did not occur. This outcome is similar to those previously described as being dependent upon the degree of severity of GO and the time from initiation of treatment to intervention [23,24].

At follow-up, the patient remained biochemically euthyroid on levothyroxine replacement therapy, although ophthalmologic improvement was only partial. Newer immunosuppressive drugs including sirolimus and teprotumumab have shown some efficacy in refractory cases of GO [25,26,27,28,29]. However, conventional corticosteroid therapy and surgery were used in this patient.

This case report has several limitations. As a single-patient observation, it does not allow causal inferences regarding the relationship between HT, GD, and PTC. Nevertheless, its rarity and detailed clinical, laboratory, imaging, and histopathological documentation provide valuable insights into this uncommon clinical triad.

## 4. Conclusions

This case highlights the diagnostic complexity of autoimmune thyroid disease when HT, GD with GO, and PTC coexist. It emphasizes that changes in the clinical course of autoimmune thyroid disease warrant careful reassessment, as additional pathology, including thyroid malignancy, may be present. Although a causal relationship cannot be inferred from a single case, this report adds to the limited literature and underscores the importance of individualized multidisciplinary management and long-term follow-up in patients with complex autoimmune thyroid disorders.

## Figures and Tables

**Table 1:** Laboratory results before and after treatment.

Parameter	Initial (February–April 2024)	Follow-Up (2025)	Reference Range
TSH	0.05 µIU/mL	2.59–4.49 µIU/mL	0.38–4.10
FT3	7.80 pg/mL	2.41 pg/mL (low-normal)	2.17–3.34
FT4	2.63 ng/dL	0.91 ng/dL	0.61–1.57
Anti-TPO	223.2 IU/mL	21.8 IU/mL	<31.5
Anti-TG	40.4 IU/mL	11.7 IU/mL	<58.5
TRAb	17.53 IU/L	-	0–1.75
CRP	6.6 mg/L	0.55–1.63 mg/L	0–6
Vitamin D	24.2 ng/mL	27.4 ng/mL	30–100
Hemoglobin	11.6 g/dL	12.2–12.4 g/dL	11–16

## Data Availability

The original contributions presented in this study are included in the article. Further inquiries can be directed to the corresponding author.

## Abbreviations

The following abbreviations are used in this manuscript:

Anti-TG	Anti-thyroglobulin antibody
Anti-TPO	Anti-thyroid peroxidase antibody
TRAb	Thyroid-stimulating hormone receptor antibody
CRP	C-reactive protein
TSH	Thyroid-stimulating hormone
Hb	Hemoglobin
FT3	Free triiodothyronine
FT4	Free thyroxine
GD	Graves’ disease
GO	Graves’ orbitopathy
HT	Hashimoto thyroiditis
PTC	Papillary thyroid carcinoma
R0	Complete tumor resection with negative margins
MRI	Magnetic resonance imaging
PTH	Parathyroid hormone
